# Solution Structure of the QUA1 Dimerization Domain of pXqua, the *Xenopus* Ortholog of Quaking

**DOI:** 10.1371/journal.pone.0057345

**Published:** 2013-03-08

**Authors:** Muzaffar Ali, R. William Broadhurst

**Affiliations:** Department of Biochemistry, University of Cambridge, Cambridge, United Kingdom; University of Oulu, Finland

## Abstract

The STAR protein family member Quaking is essential for early development in vertebrates. For example, in oligodendrocyte cells it regulates the splicing, localization, translation and lifetime of a set of mRNAs that code for crucial components of myelin. The Quaking protein contains three contiguous conserved regions: a QUA1 oligomerization element, followed by a single-stranded RNA binding motif comprising the KH and QUA2 domains. An embryonic lethal point mutation in the QUA1 domain, E48G, is known to affect both the aggregation state and RNA-binding properties of the murine Quaking ortholog (QKI). Here we report the NMR solution structure of the QUA1 domain from the *Xenopus laevis* Quaking ortholog (pXqua), which forms a dimer composed of two perpendicularly docked α-helical hairpin motifs. Size exclusion chromatography studies of a range of mutants demonstrate that the dimeric state of the pXqua QUA1 domain is stabilized by a network of interactions between side-chains, with significant roles played by an intra-molecular hydrogen bond between Y41 and E72 (the counterpart to QKI E48) and an inter-protomer salt bridge between E72 and R67. These results are compared with recent structural and mutagenesis studies of QUA1 domains from the STAR family members QKI, GLD-1 and Sam68.

## Introduction

**Figure 1:**
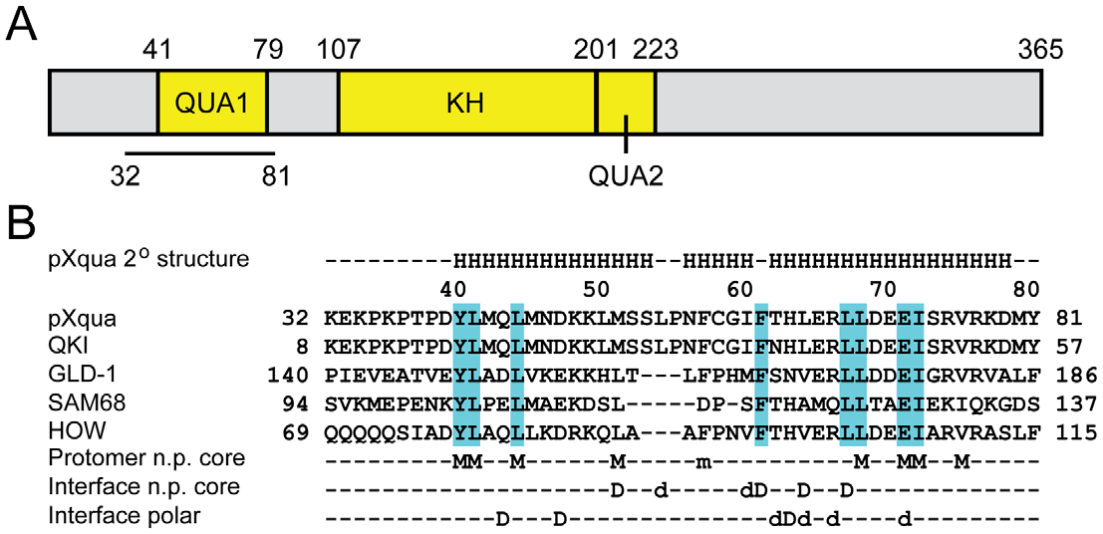
Domain organization and sequence alignments. (A) Domain organization of pXqua [16], showing location of QUA1, KH and QUA2 regions in yellow blocks, and the boundaries of the QUA1 construct used in this work as a black line below. (B) Sequence alignment and residue numbering for QUA1 regions of different STAR family members. Above, secondary structure observed for the pXqua QUA1-C59S dimer. Below, residues that contribute to the protomer non-polar core, along with non-polar and polar residues that participate in the dimer interface: capital letters indicate involvement in all known structures; small case indicates involvement only in pXqua; M and m indicate involvement in the protomer core; D and d indicate involvement in the dimer interface.

STAR (signal transduction and activation of RNA) proteins coordinate cell cycle and differentiation events in metazoa by regulating aspects of RNA metabolism that include alternative splicing, export from the nucleus, mRNA localization, and repression of translation [1]–[3]. Typical family members contain a STAR domain that spans three contiguous conserved regions: a Type I maxi-KH module and a helical QUA2 region, which together form a sequence-specific binding platform for single-stranded RNA, preceded by a QUA1 dimerization element (Figure 1A) [4]. The natively unfolded C-terminal region contains a collection of signalling motifs: proline-rich segments that can interact with WW or SH3 domains [1]; tyrosine-rich sequences that can be phosphorylated by tyrosine kinases prior to interaction with SH2 domains [5]; and arginine/glycine-rich regions that can be methylated by protein arginine N-methyltransferases [6]. Serine/threonine phosphorylation [7], [8], lysine acetylation [9] and SUMOylation [10] sites offer further opportunities for fine tuning the function of this class of adaptor proteins.

The STAR family member Quaking (termed QKI in mice and pXqua in *Xenopus laevis*) is highly conserved in multicellular eukaryotes and plays important roles in embryogenesis; in the development of glial, muscular, vascular and colon epithelial cells; and in apoptosis [11]–[16]. In mice, myelinating oligodendrocyte cells express three main alternatively spliced isoforms of QKI: QKI-5, which harbors a C-terminal nuclear localization signal; and the predominantly cytoplasmic truncated variants QKI-6 and QKI-7 [13]. Quaking Viable (*qk^v^*) mutant mice, which are afflicted by rapid tremors due to a lack of myelin in the central nervous system [17], possess a deletion in chromosome 17 that affects the promoter and enhancer regions of the *QKI* gene and suppresses production of QKI-6 and QKI-7 in oligodendrocytes [13], [18]. In *qk^v^* mice, the QKI-5 isoform binds to elements in the 3′-untranslated region of myelin basic protein (MBP) mRNA, sequestering it within the nucleus, preventing it from being exported to the cytoplasm and thereby inhibiting the expression of MBP and the production of myelin [19]. The *qk^v^* hypomyelination phenotype can be rescued by selective expression of QKI-6 in oligodendrocyte cells [20], which facilitates the export of MBP mRNA; contributes to silencing its translation and protecting it from degradation in ribonucleoprotein granules; and promotes interactions with the cytoskeleton that enable trafficking of the mRNA to the site of myelin synthesis in the distal processes [5], [21], [22]. Consistent with this picture, the expression of QKI isoforms in glial cells is temporally regulated: QKI-5 appears early in embryonic development and decreases shortly after birth, whereas QKI-6 and QKI-7 levels peak later, during myelinogenesis [13]. In the case of pXqua, two isoforms have been found, corresponding to murine QKI-5 and QKI-6 [23].

RNA interference knockdown experiments coupled with microarray analysis have suggested that QKI regulates an extensive network of transcripts [24]. Transcriptome-wide cross-linking and immuno-precipitation studies followed by deep sequencing have confirmed that a subset of these targets interact directly with QKI [25]. For example, during embryogenesis, QKI promotes the differentiation of oligodendrocyte precursor cells by protecting the mRNA that codes for cyclin-dependent kinase inhibitor p27^Kip1^ from degradation [19]. QKI also coordinates widespread events in a less direct manner by manipulating the levels of key splicing factors and micro RNAs [24], [26], [27].

*In vitro* studies employing SELEX, gel mobility shift and fluorescence polarization assays have established that a STAR binding element (SBE) hexamer with consensus sequence 5′-NA(A>C)U(A>C)A-3′ is sufficient for high affinity binding to QKI [28]–[31]. In common with other STAR proteins, the target sequence is recognized only when presented in a loop, not in double stranded RNA [32], [33], and multiple SBEs can work in concert to increase affinity for QKI [31], [34]. Taken together, these results imply that QKI homodimers may bind two SBEs on the same RNA molecule simultaneously, or may recruit multiple RNAs into a ribonucleoprotein particle. Interestingly, a point mutation in the QUA1 region (E48G) that disrupts the dimerization of QKI was found to be embryonically lethal in mice [35]. By contrast, deleting the QUA1 domain of the *C. elegans* homolog GLD-1 decreased its affinity for RNA by an order of magnitude, but did not abolish binding completely [36].

Our previous nuclear magnetic resonance (NMR) spectroscopy studies defined a β1−α1−α2−β2−α3−α4−β3−α5−α6 topology for a protein fragment spanning the KH and QUA2 domains of *Xenopus* pXqua and highlighted the importance of nascent structure in the QUA2 portion in the absence of RNA [37]. In this report we investigate the solution structure of the adjacent QUA1 domain of pXqua, comparing the results with recent studies of dimerization motifs in other STAR family members [38]–[40] and exploring why QKI E48G mutants fail to dimerize.

## Experimental Procedures

### Protein Expression and Purification

Using the full length *Xenopus pXqua* gene carried by plasmid pGEX-4T-3-pXqua [37] as a template, the sequence coding for residues 32 to 81 of pXqua was cloned into a pMAT10 expression vector and expressed in *Escherichia coli* BL21(DE3)-pLysS cells. Point mutations were introduced into pMAT10-QUA1 via the QuikChange protocol (Stratagene), using the primers detailed in the Supporting Information, Tables S1, S2, S3, S4, S5 in File S1. The protein products included an N-terminal His_6_-maltose binding protein (MltBP) double affinity tag, followed by a thrombin recognition sequence. After cleavage of the fusion protein, all QUA1 constructs contained two additional residues (GS) at their N-termini. The identity and molecular weight of all protein samples were confirmed using electrospray mass spectrometry on a Q-Tof micro system (Waters).

For preparation of unlabelled samples, bacteria were grown in LB medium. For uniformly double labeled samples, 1X MOPS minimal medium supplemented with ^15^NH_4_Cl (final concentration: 10 mM), ^13^C-glucose (20%) and Celtone-CN (2%, Martek) was used. After induction of protein expression with 1 mM isopropyl-1-β-thiogalactoside (IPTG), cells were incubated at 20°C for 20 h. Protein constructs were enriched using a Nickel-NTA column (Qiagen), eluted, cleaved with thrombin (for QUA1 samples) and then purified via amylose resin affinity chromatography (BioRad) followed by 16/60 Superdex-75 size exclusion chromatography (GE Healthcare). The purity of fusion protein and released peptide products was confirmed by SDS-PAGE analysis (Figure S1 in File S1). An asymmetrically labeled [^12^C,^14^N]/[^13^C,^15^N] sample was prepared by mixing equimolar amounts of unlabeled and ^15^N/^13^C uniformly labeled QUA1 in a buffer containing final concentrations of 50 mM sodium phosphate and 100 mM sodium chloride, followed by incubation at 70°C for 5 min and slow cooling back to room temperature.

### Analytical Size Exclusion Chromatography

Pure samples were analyzed on Superdex-75 PC 3.2/30 (2.41 mL) and Superdex-200 PC 3.2/30 (2.41 mL) analytical size exclusion columns using an ETTAN LC system (Amersham Biosciences) and a buffer solution containing Tris-HCl (final concentration 20 mM, pH 7.9), sodium chloride (150 mM) and sodium azide (0.05% v/v). The columns were initially calibrated by running a set of standard protein samples of known molecular weights. *K*_av_ was calculated using the equation: *K*_av_ = (*V*_e_ – *V*_o_)/(*V*_t_ – *V*_o_), where *V*_e_ represents the maximum of the experimental elution profile, *V*_t_ the total column volume, and *V*_o_ the void volume. 50 µL protein samples at a concentration of 1 mg mL^–1^ were loaded via a loop and the column was operated at a flow rate of 50 µL min^–1^ with a fraction size of 80 µL. Elution profiles were monitored by measuring absorbance at 280 nm. Protein apparent molecular weights (AMW) were estimated from linear calibration plots of *M*_r_^1/3^ against (–log_10_(*K*_av_))^1/2^ [40]. The following relationships were used to predict the apparent hydrodynamic (Stokes) radius *R*_S_ for different forms of the pXqua QUA1 domain: for globular folded protein, log_10_(*R*_S_/Å) = –0.204+0.357 × log_10_(*M*_r_/Da); and for unfolded protein, log_10_(*R*_S_/Å) = –0.551+0.493 × log_10_(*M*_r_/Da) [42]. Apparent Stokes radii of protein samples were estimated from linear calibration plots of *R*_S_ against (−log_10_(*K*_av_))^1/2^ [41]. Crude estimates of the fraction of dimeric MltBP-QUA1 fusion protein present in mixtures were obtained from the ratio (*R*_S,obs_ – *R*_S,monomer_)/(*R*_S,dimer_ – *R*_S,monomer_); *R*_S,dimer_ was set to 44 Å, and *R*_S,monomer_ to 32 Å.

### NMR Spectroscopy

All NMR samples were prepared at a protein concentration of 1 mM in a buffer containing final concentrations of 50 mM sodium phosphate and 100 mM sodium chloride at pH 6.0, supplemented with 0.05% (w/v) sodium azide, 20 µM 3,3,3-trimethylsilylpropionate and 10% D_2_O, to a final volume of 550 µL in 5 mm Ultra-Imperial grade NMR tubes (Wilmad). Spectra were recorded at 298 K on Bruker DRX500 and DRX800 spectrometers equipped with z-shielded gradient triple resonance probes. [^1^H, ^15^N]-HSQC, ^15^N-TOCSY-HSQC, ^15^N-NOESY-HSQC, ^13^C-NOESY-HSQC, HNCA, HN(CO)CA, HNCACB, CBCA(CO)NH, HNCO, HBHA(CO)NH, HCCH-TOCSY and ^15^N-relaxation spectra were recorded using standard procedures [43]. Inter-molecular contacts were obtained from a ^13^C/^15^N X-filtered NOESY experiment on a [^12^C,^14^N]/[^13^C,^15^N] asymmetrically labelled sample [44]. ^1^H-^15^N residual dipolar couplings were collected in an alignment medium containing 3% C_12_E_5_/hexanol (molar ratio = 0.96) at pH 6.0 [45]. Solvent ^2^H quadrupolar splitting measurements indicated that the medium was aligned at 308 K and isotropic at 303 K. For further details of acquisition and analysis, see the Supporting Information File S1.

### Structure Determination

All structures were calculated from extended templates by simulated annealing using ARIA version 2.3 [46], with manual screening of ambiguous restraints. Backbone φ and ψ dihedral angle restraints were determined from chemical shifts using the DANGLE program [47]. Restraint lists generated by the resonance assignment process were fed as input and nine iterations were performed, each using 40 structures, except for the final round, in which 80 were calculated, followed by refinement in explicit solvent for the 40 lowest energy structures. The 20 lowest energy refined structures with no NOE violations greater than 0.5 Å and no dihedral angle violations greater than 5° were selected for the final ensemble. Structural alignments and buried surface area measurements were obtained using the Smolign [48] and PDBePISA (http://www.ebi.ac.uk/msd-srv/prot_int/pistart.html) servers, respectively. Figures were generated using PyMOL (http://pymol.sourceforge.net/).

### Accession Codes

Protein Data Bank: the atomic coordinates of the final, together with the experimental distance and dihedral angle constraints, were deposited under wwPDB ID code 2YMJ. Biological Magnetic Resonance Data Bank: the NMR assignments were deposited under accession code 18782.

## Results

### Oligomeric State of the pXqua QUA1 Domain

Prior studies of wild type and mutant fragments of GLD-1, SAM68 and QKI had concluded that monomeric forms of the QUA1 domain are unfolded [36], [39], [40]. Based on expected molecular weights, we estimated that the apparent hydrodynamic radii (*R*_S_) of unfolded monomeric and entirely folded globular dimeric forms of the pXqua QUA1 domain should be 21 Å and 18 Å, respectively [42]. Any unstructured residues at the termini of our pXqua QUA1 construct would likely increase the apparent *R*_S_ value of the dimeric state, so we judged that analytical size exclusion chromatography (SEC) would fail to discriminate unambiguously between folded and unfolded forms of the domain. We therefore used SEC to investigate N-terminal fusions of maltose binding protein (MltBP) to the pXqua QUA1 domain, adapting procedures developed by Ryder and co-workers for determining the oligomeric state of GLD1 MltBP-QUA1 fusion proteins [36]. This approach depends on the larger, intrinsically monomeric MltBP partner remaining structured independent of the folding status of the covalently-linked pXqua fragment. Dimeric and monomeric forms of pXqua MltBP-QUA1 fusions were predicted to possess *R*_S_ values of 38 Å and >29 Å, respectively, suggesting that SEC experiments on fusion protein constructs might be capable of identifying the oligomeric state with higher accuracy than studies on released QUA1 peptides.

**Figure 2:**
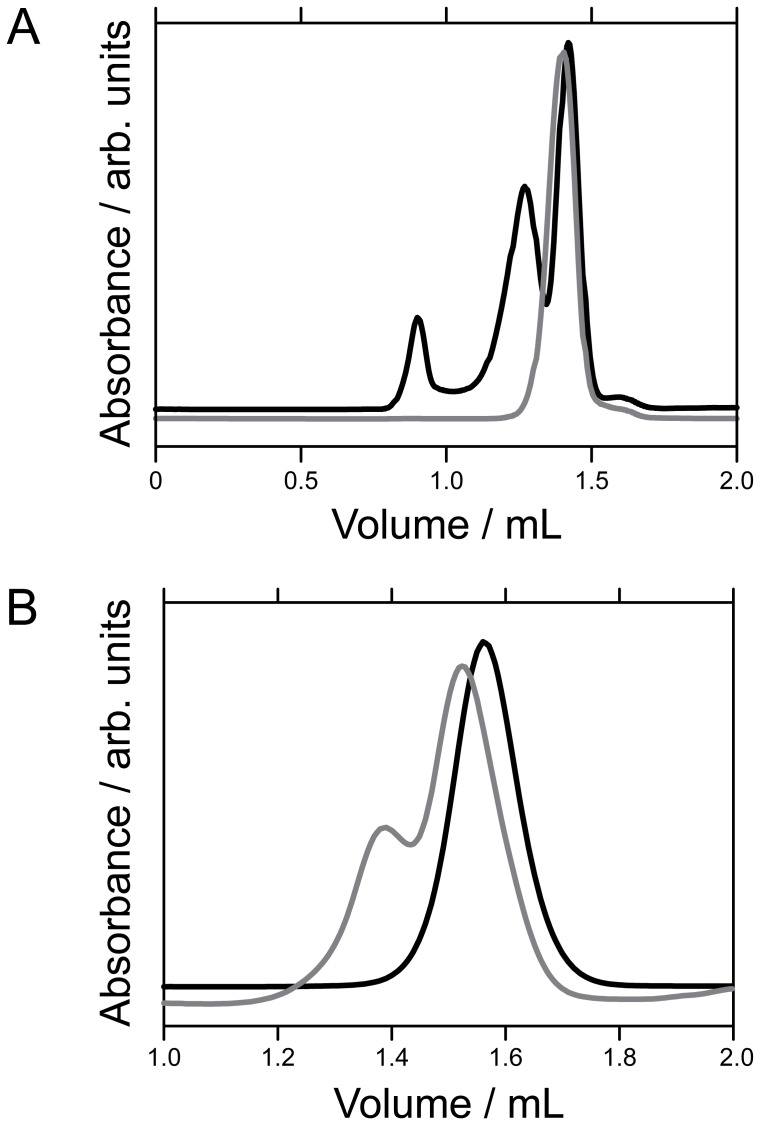
Size exclusion chromatography studies. Typical analytical size exclusion chromatography profiles, using a Superdex S200 PC 3.2/30 column and a 20 mM Tris-HCl and 150 mM NaCl running buffer at pH 7.9, for: (A) wild type pXqua MltBP-QUA1 (black) and MltBP-QUA1-C59S (grey); and (B) MltBP-QUA1-C59S/E72G (black) and MltBP-QUA1-C59S/R67A/E72G (grey).

Wild type MltBP-QUA1 fusion protein eluted from a Superdex S200 column in two fractions at 1.27 mL and 1.47 mL (Figure 2A), corresponding to apparent hydrodynamic radii of 54 Å and 42 Å, apparent molecular weights (AMWs) of 234 kDa and 120 kDa, and apparent aggregation numbers (AANs) of 4.8 and 2.4, respectively (Table 1). Because the wild type pXqua sequence contains a cysteine residue at position 59, we reasoned that exposure to atmospheric oxygen might produce a mixture of dimers and non-native disulphide cross-linked tetrameric species. The presence of intermolecular disulphide bonds in the larger species was verified by SDS-PAGE in the presence and absence of the reducing agent 1,4-dithio-D-threitol (Supporting Information, Figure S2 in File S1). We used site-directed mutagenesis to prepare a MltBP-QUA1-C59S mutant, which eluted from the column in a single fraction at 1.40 mL (Figure 2A), corresponding to an *R*_S_ value of 44 Å, an AMW of 131 kDa and an AAN of 2.7. The discrepancy between the molecular weight expected for this construct (96 kDa) and the larger AMW measured by SEC likely occurs because the shape of the fusion protein dimer deviates from a rigid sphere. Further SEC experiments on the protease-liberated QUA1-C59S peptide using a Superdex S75 column yielded an AAN value of 2.6 and an apparent *R*_S_ of 19 Å (data not shown), intermediate between the predicted values of 21 Å for an unfolded monomer and 18 Å for a folded dimer. All subsequent experiments were performed using C59S mutant forms of the pXqua QUA1 domain. The association state of released QUA1-C59S was validated using analytical ultracentrifugation: global single species fits to sedimentation equilibrium data (see Supporting Information, Figure S3 in File S1) yielded a mean molecular weight of 12.2±0.3 kDa, which matched the theoretical value of 12.3 kDa expected for a folded dimer. Taken together, these results confirm that the QUA1 region of pXqua is a homo-dimerization motif.

**Table 1:** Interpretation of analytical size exclusion chromatography results for C-terminal fusions to maltose binding protein (MltBP) of pXqua QUA1 domain mutants.

Sample	Elution volume/mL	AAN^a^	Oligomeric state
MltBP-QUA1	1.27/1.42	4.8/2.4	Tetramer/Dimer
MltBP-QUA1-C59S	1.40	2.7	Dimer
MltBP-QUA1-C59S/E72G	1.57	1.2	Monomer
MltBP-QUA1-C59S/R67A	1.50	1.7	Monomer/Dimer
MltBP-QUA1-C59S/R67A/E72G	1.39/1.53	2.8/1.5	Monomer/Dimer
MltBP-QUA1-C59S/R67E/E72R	1.46	2.1	Monomer/Dimer

a AAN, apparent aggregation number.

### Solution Structure of pXqua QUA1-C59S

**Figure 3:**
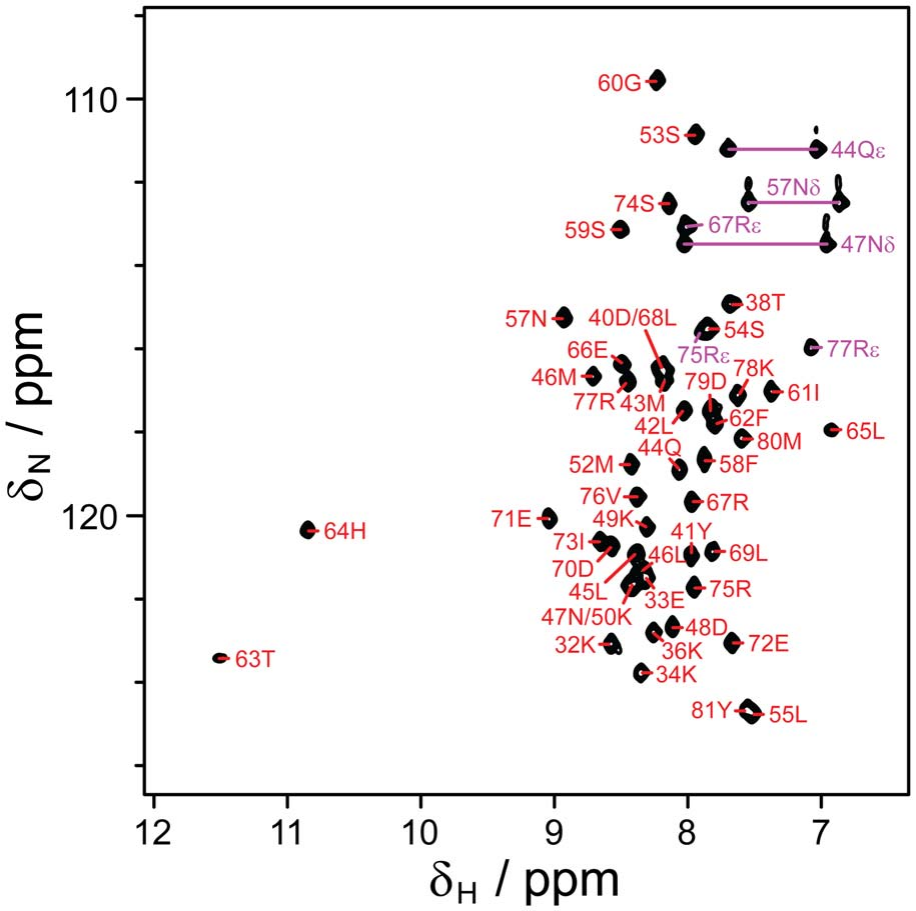
NMR spectroscopy. [^1^H,^15^N]-HSQC spectrum of pXqua QUA1-C59S, showing residue assignments for backbone amide and selected side-chain sites.

**Figure 4:**
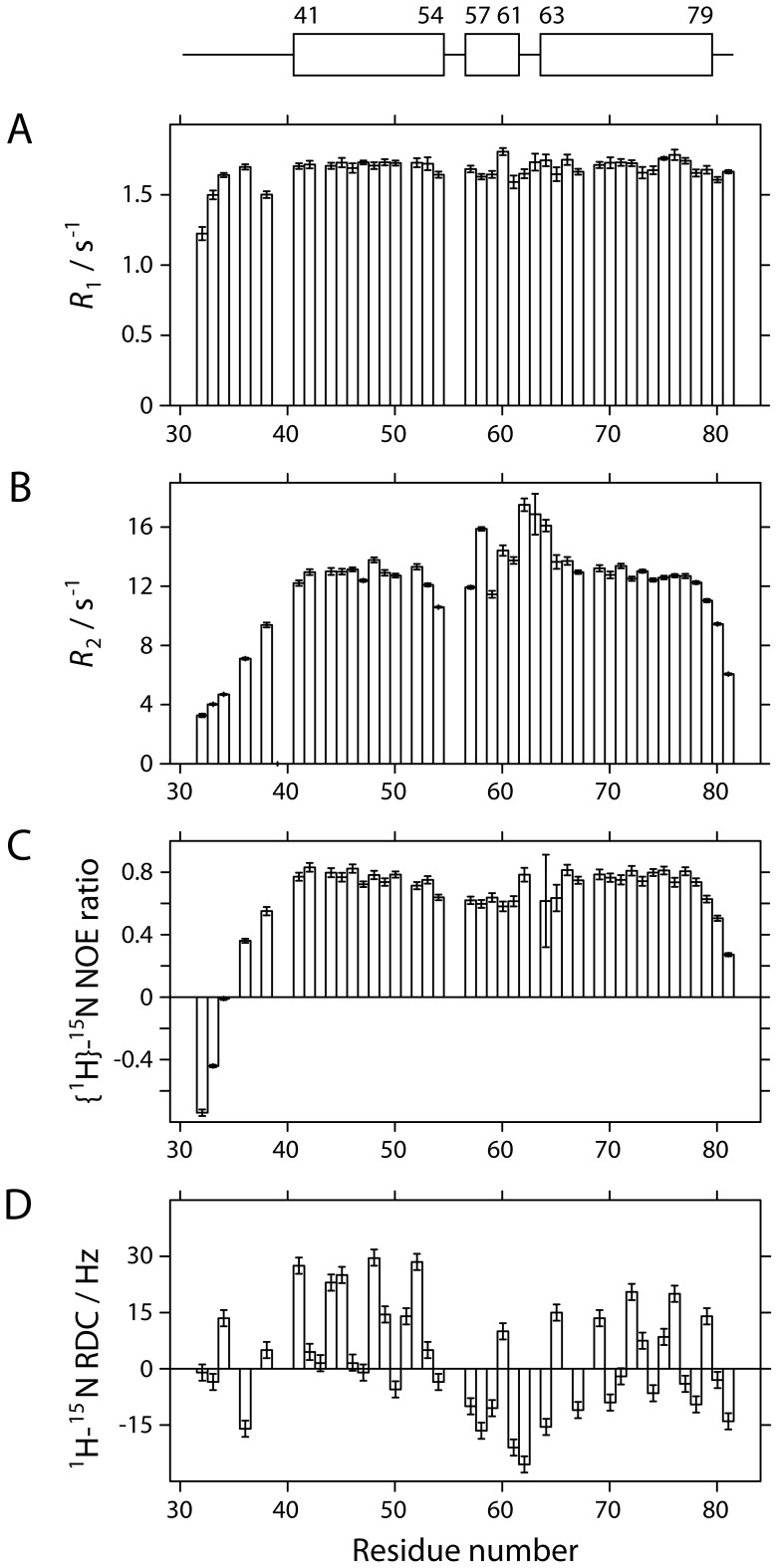
^15^N relaxation parameters. Underneath a schematic defining the boundaries of α-helices in the structure of pXqua QUA1-C59S, NMR parameters for backbone amide sites are plotted as a function of residue number for: (A) the ^15^N longitudinal relaxation rate, *R*_1_; (B) the ^15^N transverse relaxation rate, *R*_2_; (C) the {^1^H}-^15^N heteronuclear Overhauser effect ratio (*I’*/*I*_0_, where *I’* is the intensity when the ^1^H spectrum has been saturated and *I*_0_ is the intensity in the reference spectrum); and (D) ^1^H-^15^N residual dipolar coupling measurements.

The [^1^H,^15^N]-HSQC spectrum of QUA1-C59S contained 46 of the 47 expected backbone amide signals (Figure 3) and possessed a ^1^H^N^ chemical shift dispersion of 4.6 ppm, which is significantly larger than the <0.8 ppm spread expected for an unstructured polypeptide. Nearly complete assignments for resonances from backbone and non-exchangeable side-chain nuclei were obtained using a suite of standard triple-resonance NMR experiments [43]. ^15^N *R*_1_, *R*_2_ and {^1^H}-^15^N NOE relaxation data profiles (Figure 4) indicated that the structured region spans 39 residues, from Y41 to D79. An overall rotational correlation time *τ*_C_ of 9.9±0.7 ns estimated from the *R*_1_ and *R*_2_ values of well structured residues was consistent with the molecular weight expected for a dimer. Analysis of ^1^H^α^, ^13^C^α^, ^13^C^β^, ^13^C′ and ^15^N chemical shifts using the DANGLE algorithm [47] detected two α-helices, α1 (Y41 to S54) and α2 (T63 to D79), connected by a coiled turn. This secondary structure classification agreed well with short- and medium-range distance restraints identified in ^13^C- and ^15^N-separated NOESY-HSQC experiments (see Supporting Information, Figure S4 in File S1).

**Figure 5:**
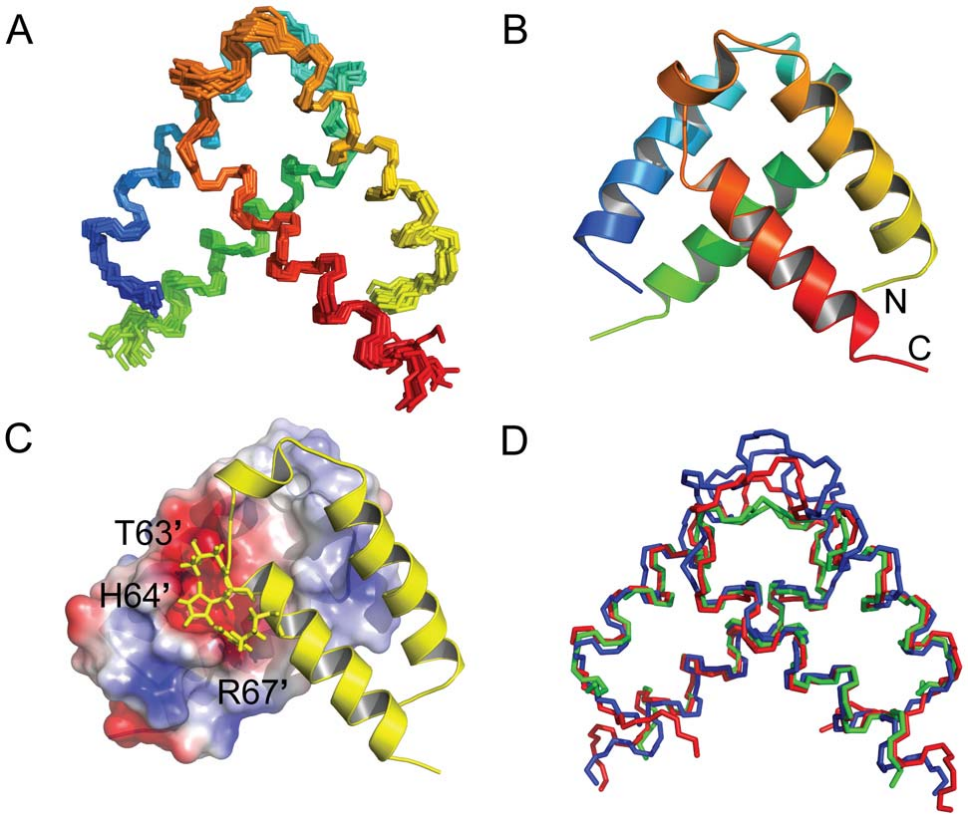
Solution structure of the of pXqua QUA1-C59S dimer. (A) backbone overlay for the final ensemble of 20 lowest energy structures, with protomer A coloured from blue (NT) to green (CT) and protomer B from yellow (NT) to red (CT); (B) ribbon representation of the dimer structure; (C) representation showing the surface of protomer A, coloured according to charge from blue (positive) to red (negative), and protomer B in ribbon form with selected residues displayed as sticks; (D) backbone overlay of QUA1 dimerization domain structures for pXqua C59S (blue), GLD-1 (3K6T; red) and Sam68 (2XA6; green).

The solution structure of the pXqua QUA1 homodimer was determined using 1403 NOE distance restraints, including 21 inter-protomer distances derived from a ^12^C/^14^N-filtered ^13^C-separated NOESY-HSQC experiment performed on an asymmetrically [^12^C,^14^N]/[^13^C,^15^N]-labelled protein sample. Further details of the restraints and structural statistics are summarized in Table 1. After water refinement, the final ensemble comprised the 20 lowest energy structures with no distance violations greater than 0.5 Å and no dihedral angle violations greater than 5° (Figure 5A). Each protomer folds into an α-helical hairpin, with the two helices meeting at an angle of 31±2°, elaborated in the connector region by a short additional helix between residues N57 and I61 (Figure 5B). The hairpin motif is stabilized by van der Waals contacts between the non-polar side-chains of L42, L45 and M52 in α1 and L69, I73 and V76 in α2, capped by interactions with the aromatic ring of F58 from the connector region and pinned by a hydrogen bond between the side-chains of Y41 and E72 (Figure 1B).

The heads of two hairpin motifs dock at an angle of 85±3° to create a dimer interface. This interaction is mediated by non-polar contacts between the side-chains of M52, L55, I61, F62, L65 and L68; by interactions between the aromatic side-chains of Y41 and H64′ (in the facing protomer); by main-chain/side-chain hydrogen bonds from T63 and H64 to D48′; by side-chain/side-chain hydrogen bonds from H64 to Q44′; and by electrostatic interactions between the side-chains of D48 and H64′, E72 and R67′ (Figure 1B). Most of the interface is non-polar, but D48 and E72 line a negatively-charged cavity that accommodates the positively-charged side-chains of H64′ and R67′ (Figure 5C). Large downfield chemical shifts observed for the amide ^1^H^N^ resonances of T63′ and H64′ (11.50 and 10.85 ppm, respectively; Figure 3) confirm the presence of hydrogen bonds to the side-chain carboxyl group of D48.

Between Y41 and D79, the models in the final ensemble superimpose closely, yielding a root mean square deviation (RMSD) for dimer backbone atom coordinates of 0.67±0.15 Å (Table 2). Although this result suggests that the overall structure is rigid, atypically rapid ^15^N transverse relaxation rates observed for residues F58, F62, T63 and H64 (Figure 4B) indicate the presence of a millisecond timescale exchange process between multiple local conformations. Dynamics of this sort may be the result of minor backbone rearrangements in the connector region, or of side-chains adopting different rotameric states. For example, reorientation of the side-chain of D48 would affect the geometry of hydrogen bonds to the backbone amide sites of T63′ and H64′, leading to time-dependent chemical shift changes and consequent line-broadening effects.

**Table 2:** Restraints and statistics for the pXqua QUA1-C59S homodimer solution structure.

NOE-based distance restraints	Number
Intra-residue, sequential	804
Medium range (2 ≤ | *i* – *j* | ≤ 5 )	314
Long range (| *i* – *j* | >5 )	86
Inter-protomer	160
Ambiguous	39
Total	1403
**Other restraints**	
Hydrogen bond restraints	23
*φ*+*ψ* dihederal angle restraints	64
Residual dipolar coupling restraints (^1^H^N^-^15^N RDCs)	37
**Coordinate precision** a	
Protomer backbone r.m.s.d. (Å)	0.55±0.12
Protomer heavy atom r.m.s.d. (Å)	1.21±0.14
Dimer backbone r.m.s.d. (Å)	0.67±0.15
Dimer heavy atom r.m.s.d. (Å)	1.28±0.14
**Consistency (structure vs restraints)**	
R.m.s.d. (Å) from experimental distance restraints	0.021±0.005
R.m.s.d. (°) from experimental dihedral angle restraints	0.4±0.2
R.m.s.d. (Hz) from experimental ^1^H^N^-^15^N RDC restraints	0.46±0.04
**Ramachandran plot** a	
Most favoured regions	93.0%
Allowed regions	5.7%
Generously allowed regions	1.3%
Disallowed regions	0.0%
**WHATIF structure Z-scores** a	
First generation packing quality	3.185±0.594
Second generation packing quality	5.235±1.124
Ramachandran plot appearance	–3.503±0.410
χ_1_/χ_ 2_ rotamer normality	–3.539±0.672
Backbone conformation	–0.255±1.038

a Coordinate precision, Ramachandran statistics and Z-scores were determined between residues Y41 and D79.

### The Effect of Mutations on Dimer Stability

We explored the deleterious effects of the QKI E48G point mutation by characterizing apparent aggregation numbers for mutant forms of the pXqua QUA1 domain. A MltBP-QUA1-C59S/E72G double mutant fusion construct eluted from the SEC column in a single fraction at 1.57 mL (Figure 2B), corresponding to an apparent hydrodynamic radius of 32 Å and an AAN of 1.2, showing that dimerization had not occurred. Further SEC experiments on the released QUA1-C59S/E72G peptide yielded a *R*_S_ value of 20 Å and an AAN value of 2.9 (data not shown). These results confirm that monomeric states of the QUA1 domain are unfolded and that the released peptide constructs exhibit very similar hydrodynamic properties to the folded dimeric form (*R*_S_ 19 Å).

The wild type sequences of helices α1 and α2 possess very low propensities for independent folding: the highest predicted populations for α1 and α2 are 9% and 12%, respectively (Supporting Information, Figure S5B in File S1). These low values suggest that the folding of the QUA1 domain is not governed by the docking of stable elements of secondary structure, but rather depends on the formation of higher order structure. Further, the simple two-state transition observed for thermal denaturation of QUA1-C59S (see Supporting Information, Figure S6 in File S1) implies that globular monomer forms of the QUA1 domain are unstable, so that disruption of the quaternary structure generates an unfolded monomeric state without populating folded monomeric intermediate states. The helix breaking effect anticipated for an E72G mutation causes a further reduction in the helical propensity of α2, reducing its predicted population to 4% (Supporting Information, Figure S5C in File S1). This additional destabilization could be regarded as a minor perturbation in a region that is already unlikely to fold independently, so we focussed instead on tertiary and quaternary interactions that would be disrupted in a mutant dimer.

In the pXqua QUA1 structure, the side-chain of E72 is buried at the periphery of the dimer interface, making intra-molecular contacts with the side-chains of Y41, L45 and V76 and inter-protomer connections with R67′ and L68′. To investigate the role played by electrostatic and hydrogen bonding interactions between E72 and R67′, we purified a MltBP-QUA1-C59S/R67A construct, which eluted from the SEC column at 1.50 mL, corresponding to an AAN of 1.7 (Table 2). By assuming that separate maxima would not be resolved in the elution profile of a mixture of similarly populated folded and unfolded states, and that the maximum of the overall envelope would vary linearly with the composition of the mixture (see Experimental Procedures), we estimated that approximately 40% of fusion protein species were present in the dimeric state. Our interpretation was that substitution by alanine at position 67 must break both the inter-protomer salt bridge with E72′ and an intra-molecular interaction with the side-chain of E71; electrostatic repulsion between the negatively charged side-chains of E72′ and E71 would therefore disfavour the dimeric state.

Next, we sought to ameliorate repulsive interactions between the protomers by preparing a MltBP-QUA1-C59S/R67A/E72G triple mutant; this eluted in two fractions, with approximately one third at 1.39 mL and two thirds at 1.53 mL (Figure 2B), corresponding to AANs of 2.8 and 1.5, respectively. Thus, loss of side-chain charge on both sides of the R67/E72′ salt bridge partially restores the ability of the QUA1 domain to dimerize, resulting in a mixture of monomeric and dimeric species at room temperature.

Finally, we reasoned that single-residue mutations such as R67A and E72′G would increase backbone flexibility and create cavities at the dimer interface, minimizing opportunities for van der Waals contacts with nearby non-polar side-chains (Y41′, L45′, V76′ and L68) and disfavouring dimerization. To show the importance of both electrostatic and non-polar interactions in dimer formation, we aimed to swap the polarity of the R67-E72′ salt bridge while maintaining side-chain volume by engineering a MltBP-QUA1-C59S/R67E/E72R triple mutant; this eluted in a single fraction at 1.46 mL, corresponding to a dimeric species with an AAN of 2.1 (Table 2). Using the linear approximation, we estimated that ∼70% of fusion protein species were present in the dimeric state. At the edge of the wild type protein-protein interface, the side-chains of R67 and E72′ participate in a local network of salt-bridges involving residues H64, E71 and R75. Although electrostatic and hydrogen bonding interactions clustered across an interface can promote dimer stability, these effects go hand in hand with entropically unfavourable restraints on side-chain conformation, making it difficult to predict the overall effect of mutations solely on the basis of structural information [49]. Our observation that the polarity-swapped mutant favours the stable dimeric form indicates that the QUA1 interface requires both close packing and support from a favourable electrostatic interaction between side-chains at positions 67 and 72′; the polarity of the salt bridge and its participation in a wider electrostatic network appear to be of secondary importance.

Taken together, these experiments confirm that the side-chain of pXqua E72 participates in a network of short range intra- and inter-molecular interactions, several of which are disrupted by the E48G mutation in QKI, resulting in the abrogation of dimer formation under physiological conditions.

## Discussion

The life cycle of an RNA transcript takes place within a dynamic multi-component ribonucleoprotein (RNP) complex. The composition of an RNP assembly undergoes continual remodelling: new proteins are loaded at each stage of RNA metabolism, some of which are left behind to regulate downstream events, acting for example as quality control markers that license subsequent processing steps [50], [51]. *Xenopus* pXqua and murine QKI isoforms are likely recruited to RNP complexes during assembly of the spliceosome and influence transcript export, localization and stability because they remain attached, perhaps until their RNA target is translated or degraded.

Individual RNA-binding domains (RBDs) recognize short, common sequence motifs, so specificity for complex full-length targets is usually achieved by combining the interactions of multiple RBDs connected by polypeptide linkers [52]. Because STAR proteins possess a single RBD, spanning the conserved KH and QUA2 regions [4], it is tempting to assume that the adjacent QUA1 homodimerization motif must play an important role in enhancing the specificity and affinity of their interactions with RNA. Over the QUA1 region the sequence identity between pXqua and family members QKI, GLD-1 and Sam68 is high, at 98%, 39% and 33%, respectively (Figure 1B). The folds of the four domains also bear a close resemblance: the coordinates of 72 backbone C^α^ atoms from each dimer can be superimposed with a root mean square deviation from the mean (RMSD) of 1.84 Å (Figure 5D). Their sequences differ most in the connector region of the hairpin motif (Figure 1B): this is shorter by 3 residues in the QUA1 domain of GLD-1 (which disrupts the additional short helix found between residues N57 and I61 in pXqua) and by a further 3 residues in Sam68 (a deletion accommodated by ending helix α1 two residues early). These changes reduce the surface area that becomes buried on dimer formation (850 Å^2^ for pXqua; 815 Å^2^ for QKI; 703 Å^2^ for GLD-1; and 604 Å^2^ for Sam68), which probably determines the thermal stabilities of the resulting complexes: according to circular dichroism (CD) spectroscopy, the melting temperatures (*T*_m_) are 65°C for pXqua QUA1-C59S (see Supporting Information Figure S6 in File S1); 69°C for QKI [40]; 63°C for GLD-1 [38]; and 48°C for Sam68 [39]. All four interfaces are relatively small, falling in the sub-1000 Å^2^ range expected for transient protein-protein interactions with dissociation constants between 1 µM and 1 mM [53]. However, rotational correlation time measurements on dilute Sam68 samples are consistent with *K*_D_ values <1 µM [39], so the small contact area of these interfaces more likely reflects the compact nature of the QUA1 helical hairpin fold [54].

The side-chains that participate in the non-polar core of each monomer subunit are highly conserved, although the shortened connector region of the Sam68 QUA1 domain rules out a capping contribution equivalent to that from pXqua F58 (Figure 1B). Studies of GLD-1 and QKI have established that substitution with alanine at sites equivalent to pXqua M52 and F58 moderately reduces the thermal stability of the dimer (lowering *T*_m_ by ≤ 10°C), whereas mutations at the counterparts of L42 and V76 are strongly destabilizing (Δ*T*_m_<–15°C; see Table 3). Non-polar residues corresponding to M52, F62 and L68 of pXqua participate in the interfaces of all four dimer structures (Figure 1B), but inter-molecular contacts involving L55 and I61 are again only possible because of the extended connector region in pXqua and QKI. Alanine mutagenesis has confirmed that the counterparts of pXqua F62 and L68 play important roles in the stability of the QKI, GLD-1 and Sam68 QUA1 domains (Table 3). The non-polar character of the residue equivalent to pXqua L65 is maintained in each case, but the volume of its side-chain is variable, reducing to a valine in GLD-1 (V170) and an alanine in Sam68 (A121). The bulky L65 side-chain of pXqua appears to be accommodated by complementary substitutions in the matching interface at positions D48′, M52′ and L65′. Intriguingly, a GLD-1 V170A mutant possessed slightly increased thermal stability (Table 3), suggesting that the interface around this site can relax to accommodate a range of side-chain volumes.

**Table 3:** Thermal stability of STAR protein QUA1 dimer point mutants.

Species a	Site	pXqua equivalent	*T*_m_/°C	Δ*T*_m_/°C
GLD-1	Y149F	Y41F	44	–19
Sam68	Y103S	Y41S	42	–6
QKI	Y17F	Y41F	45	–24
GLD-1	L150A	L42A	37	–26
QKI	L18A	L42A	49	–20
QKI	Q20A	Q44A	65	–4
QKI	L21A	L45A	<15	<–57
GLD-1	R156A	D48A	62	–1
Sam68	E110A	D48A	39	–9
QKI	L27A	L51A	67	–2
GLD-1	L160A	M52A	53	–10
QKI	L31A	L55A	62	–7
GLD-1	F163A	F58A	52	–11
QKI	F34A	F58A	64	–5
QKI	I37A	I61A	67	–2
GLD-1	F167A	F62A	44	–19
Sam68	F118S	F62S	<25	<–20
QKI	F38A	F62A	53	–16
GLD-1	N169A	H63A	58	–5
Sam68	H120K	H64K	<25	<–20
QKI	H40A	H64A	58	–11
GLD-1	V170A	L65A	67	+4
QKI	R43A	R67A	57	–12
GLD-1	L173A	L68A	28	–35
QKI	L44A	L68A	37	–32
QKI	L45A	L69A	40	–29
GLD-1	E177A	E72A	48	–15
QKI	E48A	E72A	40	–29
QKI	E48G	E72G	<15	<–57
QKI	I49A	I73A	38	<–31
GLD-1	V181A	V76A	46	–17
QKI	V52A	V76A	60	–9

a Data for GLD-1, Sam68 and QKI taken from [38], [39] and [40].

A prominent feature of the interface common to all four QUA1 domains is an electrostatic interaction corresponding to that between D48 and H64′ of pXqua. The carboxyl group of pXqua D48 also engages the backbone amide sites of T63′ and H64′ via inter-protomer hydrogen bonds. The other family members possess similar pairs of hydrogen bonds, but for GLD-1 and Sam68 the aspartate is replaced by a longer glutamate side-chain, which changes the register of the participating donor sites by one residue: from E156 to N169′ and V170′ in GLD-1; and from E110 to H120′ and A121′ in Sam68. Additional evidence for inter-protomer hydrogen bonding is provided by the finding that ^1^H signals for these backbone amide sites are strongly downfield shifted in all four dimers ([39], [40]; Beuck, C. and Williamson, J.R., personal communication). Despite both sets of residues remaining buried (<20% exposed to solvent), alanine mutagenesis at E110 had only a minor destabilizing effect on Sam68 and no significant changes were observed for E156A and N169A mutants of GLD-1; by contrast, a QKI H40A mutant showed a Δ*T*_m_ of –11°C, but no N39 mutants were reported (Table 3). These observations highlight the dangers of attempting to predict electrostatic contributions to protein stability solely from structural information [55].

Early ethylnitrosourea-induced mutagenesis screens of the *Quaking* locus on mouse chromosome 17 identified a lethal dysmyelination phenotype caused by an A1007G transition that resulted in an E48G mis-sense mutation in the QUA1 domain of QKI [56], [57]. Subsequent co-immunoprecipitation assays indicate that this mutation disrupted the self-association of QKI, but did not prevent it from binding RNA [58]. A later study used an *in vitro* fluorescence polarization assay to show that a QKI C35S mutant bound tightly to RNA with a dissociation constant of 5 nM, but the *K*_d_ increased more than fourfold to 22 nM when an additional E48G mutation was made [40]. We have used analytical size exclusion chromatography to confirm that E72G mutants of QUA1 fragments from pXqua are unfolded monomers that are not able to dimerize at room temperature. E72 is strongly conserved in STAR proteins (Figure 1B), so the effects of mutation at this site have been probed in other family members: CD spectroscopy demonstrated that a QKI C35S/E48G mutant has a *T*_m_ <15°C [40], whereas a GLD-1 E177A mutant was found to be stable at room temperature, but its *T*_m_ was 15°C lower than that of the wild type sequence [38]. Substitution with glycine at protein-protein interfaces is typically more destabilizing than replacement with alanine, because glycine residues can access a wider range of backbone dihedral angle conformations [59]. In accord with this, a QKI C35S/E48A mutant was significantly more stable than the glycine variant, displaying a *T*_m_ of 40°C [40].

In the pXqua QUA1 structure, the carboxylate group of E72 from helix α2 forms an intra-molecular hydrogen bond to the side-chain of the strongly conserved Y41 residue from helix α1. In GLD-1, selective deletion of this interaction by substituting the equivalent residue with phenylalanine was strongly destabilizing, reducing *T*_m_ at least 19°C [38]; a less conservative Y103S mutation in Sam68 yielded a smaller Δ*T*_m_ value (–6°C) [39]. These results show that the solvent-exposed Y41/E72 hydrogen bond plays an important role as a clamp that stabilizes the QUA1 monomer fold. However, since the side-chain of E72 also participates in a surface-exposed salt bridge with R67′, we explored the contribution of inter-protomer electrostatic interactions at the periphery of the dimer interface. A pXqua R67A mutant was only partially self-associated at room temperature; this observation is supported by the –12°C Δ*T*_m_ value measured for QKI R43A [40]. Swapping the wild type arginine and glutamate side-chains in pXqua created an R67E/E72R mutation that favoured the dimeric state of the QUA1 domain. Arginine is not universally conserved at pXqua position 67 (Figure 1B), but we suggest that the R67/E72′ salt bridge makes a significant further contribution to dimer stability, at least for pXqua, QKI and GLD-1. Alanine scanning mutagenesis has revealed that a few key “hot-spot” residues are responsible for most of the free energy of binding at protein-protein interfaces [60]. Glutamate side-chains are rarely identified as hot-spots, whereas larger residues that can engage in a mixture of non-polar and hydrogen bonding interactions, such as tryptophan, arginine and tyrosine, are common [60]. These considerations indicate that the pXqua E72G mutation prohibits crucial intra-molecular and inter-protomer interactions with Y41 and R67′ during the simultaneous folding and binding of the QUA1 dimerization motif.

Recently, thousands of QKI STAR binding elements (SBEs) from the HEK298 cell transcriptome were identified using PAR-CLIP techniques followed by deep sequencing [25]. Most of these SBEs were derived from intronic sequences, consistent with QKI acting as a splicing factor; the rest fell predominantly in 3′-untranslated regions, suggesting that they may be involved in the regulation of later events, such as translational repression. This abundance of mRNA and miRNA targets accounts for the pleiotropic nature of mutations in QKI and its links to a wide range of human diseases, which include glioma [61], colon cancer [62], schizophrenia [63], major depressive disorder [64], 6q terminal deletion syndrome [65] and ataxia [66].

*In vitro* experiments have demonstrated that a single hexameric consensus sequence is sufficient for high affinity interactions between RNA and STAR proteins, while additional upstream or downstream elements may alter the thermodynamics of binding, depending on the sequence and spacing context [30], [31]. The latter behavior must be the result of QUA1-mediated dimerization bringing together two KH-QUA2 domains [67]: when isolated in solution, the RNA-binding regions of a pXqua dimer should adopt a range of relative orientations, restricted only by the flexible 27-residue linker between M80 and Q106. However, the *in vivo* relevance of binding to bipartite RNA motifs remains unclear. For example, the STAR protein SF1 lacks a QUA1 motif, but nevertheless acts successfully as a splicing regulator [68]. Furthermore, although the E48G mutation prevents QKI from self-associating, it remains able to bind RNA [35], [40]; similarly, deleting the QUA1 domain of GLD-1 does not completely abolish RNA binding *in vitro* [36]. With these observations in mind, the lethal nature of the E48G mutation [35] implies that the QUA1 domain may possess functions beyond simply modulating the RNA-binding properties of QKI. Consistent with its role as an adaptor protein, one possibility is that dimerization of QKI could assemble a binding platform for interactions with auxiliary proteins in an RNP complex. The failure of QKI E48G mutants to dimerize could then prevent the assembly of a competent RNP or may remodel it in a way that adversely affects the fate of the passenger RNA molecule.

Immunoprecipitation studies have shown that threonine residues adjacent to the QUA1 domain of HOW(L), the *Drosophila* analog of QKI-5, can be phosphorylated by MAPK/ERK kinases. Nir and co-workers found that this stabilized the dimerization of HOW(L) and increased its affinity for RNA [8]. It is not immediately clear why the dimeric form of HOW(L) should require this mode of stabilization, since its QUA1 sequence is 59% identical to that of pXqua and all the important monomer core and interface residues are conserved (Figure 1B). Threonine phosphorylation will introduce new negative charges close by the QUA1 domain, but more experiments would be needed to explain how this could improve dimer stability; in addition, electrostatic considerations suggest that phosphorylation would be unlikely to favor direct binding to negatively charged RNA. Rather, covalent modification by MAPK/ERK kinases may recruit a new binding partner to the RNP, which could influence both dimerization and interactions with RNA. Interestingly, phosphorylation of the more distant C-terminal tyrosine cluster by Src-PTK enzymes works in the opposite direction, causing QKI-6 to dissociate from its RNA targets [5], an effect that could also be mediated by an auxiliary protein. Although no suitable interaction partners for QKI or pXqua have been identified to date, GLD-1 is known to interact with FOG-2, a component of an SCF E3 ubiquitin ligase complex that could play a part in RNP remodelling [38].

**Figure 6:**
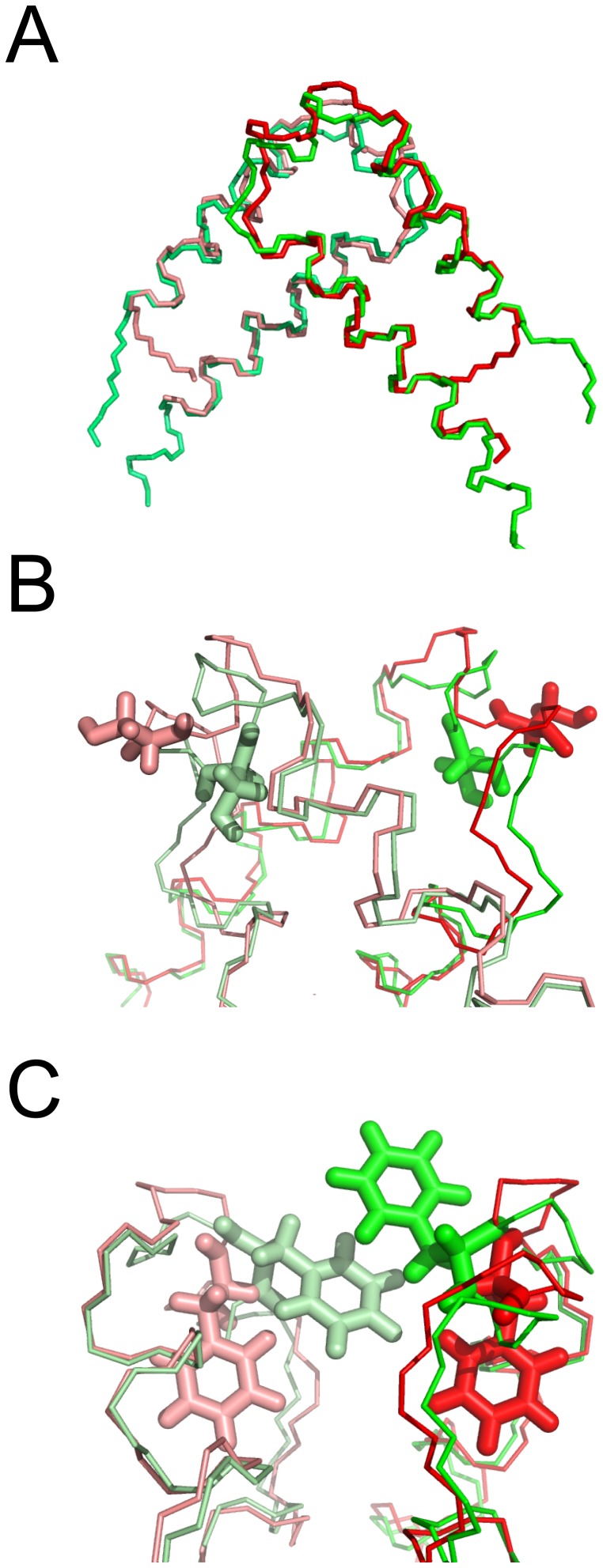
Comparison of structures of QUA1 domains from pXqua and QKI. (A) backbone overlay of dimeric QUA1 domain structures, with pXqua C59S (2YMJ) subunits displayed in red and salmon, and QKI C35S (4DNN) subunits in green and lime; (B) side-chain orientations of pXqua S59 and QKI S35, using same colours as in part (A); (C) side-chain orientations of pXqua F58 and QKI F34, using same colours as in part (A).

While this manuscript was in the final stages of preparation, the Williamson group published the X-ray structure of a C35S mutant QUA1 domain from murine QKI [40]. In general, the QKI crystal structure (PDB code: 4DNN) is very similar to the pXqua solution structure presented here: 72 backbone C^α^ atoms from each dimer superimpose with a mean RMSD of 0.65 Å (Figure 6A). The main disparities in backbone conformation are subtle changes in the hairpin connector region and an extension of helix α2 by an extra turn (although the electron density in this region is poorly defined). The C^α^ RMSD between residues P56 and T63 (pXqua numbering) is 2.24 Å; this divergence alters the extent of the connector helix: N57 to I61 for pXqua, compared with P32 to F38 for QKI. Some differences in side-chain orientation are also apparent: for example, in the QKI structure, S35 is partially buried (<36% exposed to solvent), whereas in the pXqua structure, the S59 is completely exposed (Figure 6B). The latter scenario is more consistent with our observation that the wild type pXqua QUA1 sequence readily forms disulphide-linked tetramers. Additionally, in the QKI structure the F34 side-chain protrudes from the dimer interface and engages in direct stacking interactions with its counterpart; by contrast, the aromatic side-chain of F58 is buried within the pXqua monomer structure, where it caps interactions between the non-polar side-chains of the antiparallel helical zipper motif, so it makes no inter-molecular contacts (Figure 6C). The 2.1 Å resolution QKI X-ray structure was solved using a selenomethionine derivative at 100 K, whereas the pXqua structure was determined in solution at 298 K. Our room temperature ^15^N transverse relaxation measurements highlighted a microsecond timescale conformational exchange process in the connector region of the pXqua QUA1 domain (Figure 4B), but motions of this sort would likely be restricted at lower temperatures. Minor disagreements about side-chain orientations could therefore be accounted for in terms of conformational sub-state populations becoming trapped at low temperatures. The unusual observation that the QKI F34–F34′ interaction breaks the symmetry between the two protomers [40] may have a similar explanation. We observed only a single set of H^δ^/C^δ^, H^ε^/C^ε^ and H^ζ^/C^ζ^ resonances for the side-chain nuclei of F58, rather than the two sets expected for two distinct chemical environments. However, the main conclusions of the current manuscript are strongly supported both by the crystal structure and by an accompanying analysis of QKI mutants, which highlights the contribution of a network of interactions involving the side-chains of Y17, E48 and R43 (corresponding to pXqua Y41, R67 and E72) to the overall stability of the QUA1 dimer [40].

## Supporting Information

File S1 **Supplementary experimental procedures, tables and figures.**
(PDF)
