# Supplementary material for: Solution Structure of the QUA1 Dimerization Domain of pXqua, the Xenopus Ortholog of Quaking
Source: PLoS One. 2013 Mar 8;8(3):e57345. doi: 10.1371/journal.pone.0057345 (PMC3592866; doi:10.1371/journal.pone.0057345)
Supplement: File S1 — Supplementary experimental procedures, tables and figures. (PDF) [file pone.0057345.s001.pdf]

Solution structure of the QUA1 dimerization domain of pXqua, the *Xenopus* ortholog of Quaking.

Muzaffar Ali[1,2] & R. William Broadhurst[1]

[1]Department of Biochemistry, 80 Tennis Court Road, University of Cambridge, Cambridge CB2 1GA, UK.

[2]Current address: Department of Pharmacology, University of Colorado Denver School of Medicine, Aurora, Colorado 80045, USA.

Correspondence should be addressed to R.W.B. (r.w.broadhurst@bioc.cam.ac.uk).

Supplementary Information

(1) Supplementary experimental procedures

(1.1) *Polymerase chain reaction (PCR) for sub-cloning*

PCR reactions were performed to obtain the required length of gene inserts for making various constructs using *Pfx* DNA polymerase (Invitrogen). The protocol followed for the PCR reaction was according to Table S1, with reaction condition set according to Table S2. PCR was carried out using a Triple Master PCR System (Eppendorf) following the manufacturer's instructions.

Table S1: Reaction mixture for sub-cloning PCR experiments

Step number	Reagents added	Volume / µL
1	DNA template (100 ng μL^{-1} stock)	1
2	Amplification buffer (10X)	10
3	Forward primer (125 ng μL^{-1} stock)	1
4	Reverse primer (125 ng μL^{-1} stock)	1
5	$MgSO_4$ (50 mM stock)	2
6	*Pfx* DNA polymerase (10X stock)	1
7	Enhancer solution (10X stock)	2
8	dNTPs mix (10 mM stock)	3
9	Distilled water	79
Total volume		100

Table S2: Conditions followed in sub-cloning PCR experiments				
**Step number**	**Reaction step**	**Temperature / °C**	**Duration / min**	**Number of cycles**
1	Initial denaturation	93	2	1
2	Denaturation	93	1	30
	Annealing	54	1	
	Elongation	74	1	
3	Final elongation	74	10	1
4	Suspension	4	Ω	1

(1.2) ***Site directed mutagenesis***

The overall protocol followed was adapted from the manufacturer's instructions for the QuikChange Site-Directed Mutagenesis kit (Strategene). Forward and reverse primers (Table S3) were prepared to a final concentration of 125 ng µL^{-1}. The reaction mixture was prepared according to Table S4 and PCR was performed using the conditions reported in Table S3. 1 µL of DpnI restriction enzyme was then added to the PCR reaction mixture and incubated at 37 °C for 1 h. 10 µL of the DpnI digestion products were mixed with *E. coli* DH5α competent cells, which were then transformed by the heat shock method: incubation at 42 °C for 90 s, immediately followed by incubation on ice for 2 min. Cells were then plated onto LB-amp agar plates and incubated overnight at 37 °C.

Table S3: Primer sequences	
Primer	Sequence (5'→3')
QUA1 construct:<br>pXqua QUA1-WT	FP: AAT GGG ATC CAA GGA GAA GCC GAA GCC GAC TCC AGA<br>RP: TCA TGA ATT CTC AGT ACA TAT CTT TCC GTA CTC TGC T
QUA1-C59S	FP: GCC TGC CCA ACT TCT CCG GGA TAT TTA CCC<br>RP: GGG TAA ATA TCC CGG A
QUA1-R67A	FP: CCC ACC TAG AGG CGC TCT TGG ATG AAG<br>RP: CTT CAT CCA AGA GCG CCT CTA GGT GGG
QUA1-R67E/E72R	FP: CCT AGA GGA ACT CTT GGA TGA ACG CAT CAG CAG AG<br>RP: CTC TGC TGA TGC GTT CAT CCA AGA GTT CCT CTA GG
QUA1-E72G	FP: AGA CTC TTG GAT GAA GGA ATC AGC AGA GTA CGG<br>RP: CCG TAC TCT GCT GAT TCC TTC ATC CAA GAG TCT
QUA1-E72R	FP: CTC TTG GAT GAA CGC ATC AGC AGA GTA CGG<br>RP: CCG TAC TCT GCT GAT GCG TTC ATC CAA GAG

Table S4: Reaction mixture for site directed mutagenesis experiments		
**Step number**	**Reagents added**	**Volume / µL**
1	DNA template (100 ng µL^{-1} stock)	1
2	Amplification buffer (10X)	5
3	Forward primer (125 ng µL^{-1} stock)	1
4	Reverse primer (125 ng µL^{-1} stock)	1
5	$MgSO_4$ (50 mM stock)	1
6	*Pfx* DNA polymerase (10X stock)	1
7	Enhancer solution (10X stock)	1
8	dNTPs mix (10 mM stock)	2
9	Distilled water	37
Total volume		50

Table S5: Conditions followed in site directed mutagenesis experiments				
**Step number**	**Reaction step**	**Temperature / °C**	**Duration / min**	**Number of cycles**
1	Initial denaturation	95	0.5	1
2	Denaturation	95	0.5	16-20
	Annealing	55	1	
	Elongation	68	6	
3	Final elongation	68	10	1
4	Suspension	4	Ω	1

(1.3) ***Analytical ultracentrifugation***

Sedimentation velocity (SV) and sedimentation equilibrium (SE) experiments were performed with an An60-Ti rotor in an Optima XL-1 (Beckman-Coulter) analytical centrifuge, using an Epon double-sector centrepiece and interference optics, at 20 °C. 160 µL of buffer was added into the reference section of the center-piece, while 140 µL of protein solution was added into the sample compartment at 0.5 mg mL^{-1}, 1.0 mg mL^{-1} and 2.0 mg mL^{-1} concentrations in three different cells. SV experiments were performed at 60,000 rpm. SE experiments were performed at 25,000 rpm, 36,000 rpm and 42,000 rpm with an initial equilibration period of 8 h and further equilibration period of 4 h at higher speeds. Values for the buffer density (1.00499 g mL^{-1}), viscosity (0.010214 g cm^{-1} s^{-1}) and partial specific volume of the protein sample (0.7411 mL g^{-1}) were determined using SEDNTERP [Lebowitz

et al, 2002]. All results were analyzed using SEDPHAT: SV data were fitted to a continuous sedimentation coefficient distribution model with 150 increments in the range 1.0 S to 2.0 S; and SE data were fitted globally to a single species model [Schuck, 2003].

(1.4) *NMR experiments and analysis*

Protein NMR spectra were collected using standard pulse sequences [Cavanagh et al., 2006] in a 500 MHz spectrometer (unless otherwise stated) with 128* and 1024* pairs of complex (*) points and acquisition times (τ_{max}) of 102 ms and 102 ms in the ^{15}N and ^{1}H^{N} dimensions, respectively ([^{1}H,^{15}N]-HSQC and ^{15}N-relaxation experiments); 28* × 32* × 1024* points and τ_{max} of 23 ms, 10 ms and 102 ms in the ^{15}N, ^{13}C and ^{1}H^{N} dimensions (HNCA, HN(CO)CA and HNCO); 28* × 48* × 1024* points and τ_{max} of 23 ms, 5 ms and 102 ms in the ^{15}N, ^{13}C and ^{1}H^{N} dimensions (HNCACB and CBCA(CO)NH); and 32* × 90* × 1024* points and τ_{max} of 26 ms, 13 ms and 102 ms in the ^{15}N, ^{1}H and ^{1}H^{N} dimensions (^{15}N-NOESY-HSQC); 256* × 639* points and τ_{max} of 26 ms and 64 ms in the ^{13}C and ^{1}H dimensions ([^{1}H,^{13}C]-HSQC); 30* × 64* × 1024* points and τ_{max} of 7 ms, 12 ms and 102 ms in the ^{13}C, ^{1}H and ^{1}H dimensions (HCCH-TOCSY); 31* × 88* × 1024* points and τ_{max} of 8 ms, 15 ms and 102 ms in the ^{13}C, ^{1}H and ^{1}H dimensions (^{13}C-NOESY-HSQC); 32* × 90* × 512* points and τ_{max} of 5 ms, 12 ms and 51 ms in the ^{13}C, ^{1}H and ^{1}H dimensions (^{13}C/^{15}N X-filtered NOESY; at 800 MHz). All NOESY spectra were recorded with 100 ms mixing times. Water suppression was achieved by 'flip-back' methods, using shaped selective pulses to return water magnetization to the *z*-axis prior to acquisition. Pulsed field gradients were used to suppress undesired coherence pathways and the residual water signal. All spectra were processed and interpreted using the CcpNmr Azara and Analysis packages [Vranken et al., 2005].

The ^{15}N longitudinal relaxation rate (R_1) experiment was recorded with ten delay times (10, 50, 100, 150, 250, 400, 550, 700, 850, and 1000 ms) and the transverse relaxation rate (R_2) experiment with eight delay times (14.4 (twice), 28.8, 43.2, 57.6, 86.4, 72.0, 100.8 and 115.2 ms (twice)). A heteronuclear steady state {^{1}H}-^{15}N NOE experiment was also recorded. The decay data were fit to the equation $I(t) = A.\exp(-Bt)$. For each residue the R_2/R_1 ratio was used to determine an effective rotational correlation time according to equation 8 of [Kay et al., 1989]; the mean and standard deviation of these values are reported in the main text as the overall rotational correlation time τ_C.

(1.5) *Circular dichroism*

Circular dichroism (CD) spectra were recorded with 0.1 mg mL^{-1} protein in 10 mM potassium phosphate buffer at pH 8.0 on an AVIV 315 spectrometer using quartz cuvettes. Spectra for secondary structure determination were collected between 190 nm to 250 nm. Secondary structure content was estimated using the SELCON3 algorithm on the DichroWEB server [Whitmore et al., 2004]. Thermal denaturation experiments were monitored at 209 nm, raising the temperature in 1 °C min^{-1} steps from 20 °C to 90 °C and then back to 20 °C at the same rate. Spectra were averaged over 10 scans and background spectra were subtracted as appropriate. The thermal denaturation profile was fitted to the two-state equation [Fersht, 1999]:

$$\varepsilon_{obs} = (\alpha_N + \beta_N T) + [(\alpha_D - \alpha_N) + (\beta_D - \beta_N)T] \times \exp\{(T\Delta S - \Delta H)/RT\} / [1 + \exp\{(T\Delta S - \Delta H)/RT\}]$$

where α_N, β_N, α_D and β_D describe the partial differential ellipticities of the native and denatured states, T is the absolute temperature, ΔS is the entropy of unfolding and ΔH is the enthalpy of unfolding. The melting temperature T_m was estimated from $\Delta H/\Delta S$.

(2) Supplementary results

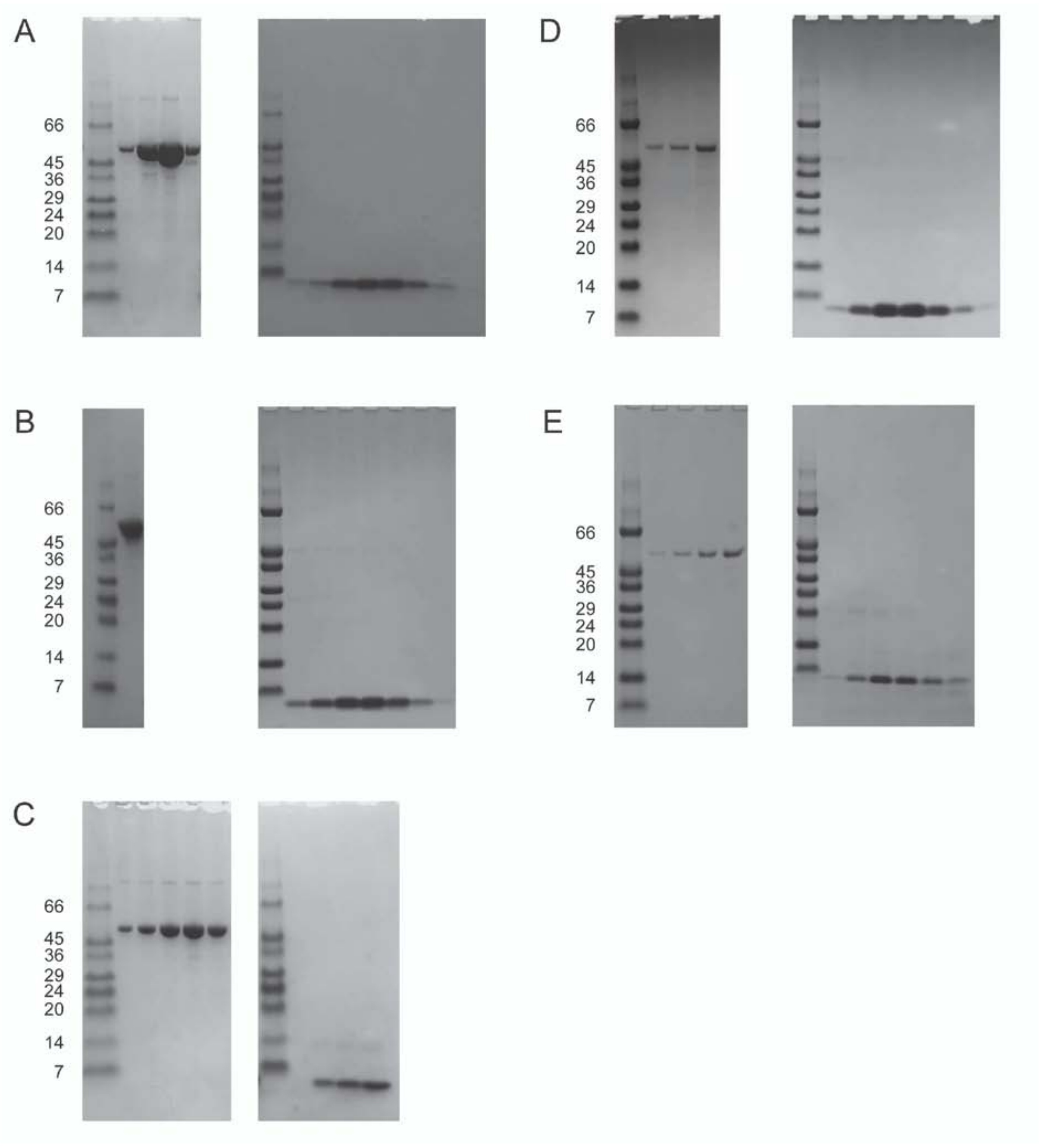

Figure S1: SDS-PAGE analysis of gel filtration fractions for various QUA1 constructs, illustrating the purity of the protein products. Results for MltBP-QUA1 fusion proteins are shown on the left and for liberated QUA1 peptides on the right: A, wild type sequence; B, C59S mutant; C, C59S/E72G double mutant; D, C59S/R67A double mutant; E, C59S/R67E/E72R triple mutant. Expected molecular weights are 48 kDa and 6 kDa for fusion proteins and peptides, respectively. All lanes denoted M contain the same set of molecular weight markers (labelled on the left in kDa).

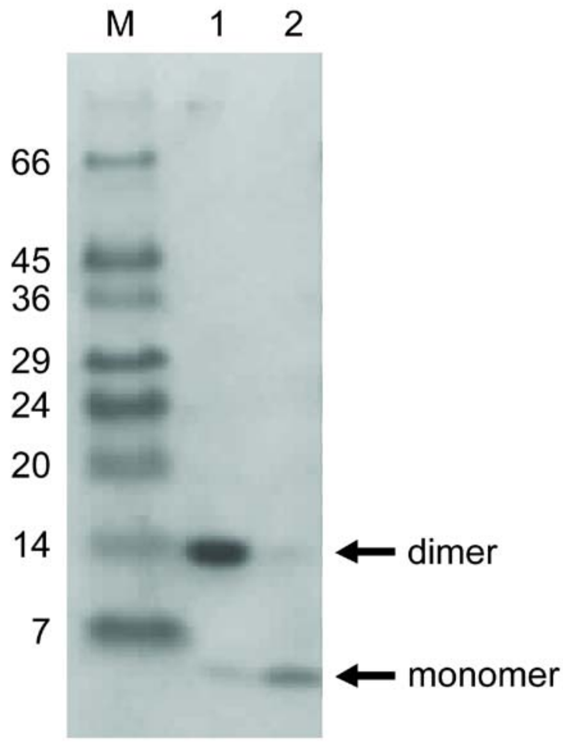

Figure S2: SDS-PAGE analysis of released wild type pXqua QUA1 fragment. Lane M contains molecular weight markers (labelled in kDa). Lane 1 contains protein boiled in the presence of loading buffer (containing: 100 mM Tris-HCl, pH 6.8; 4 % w/v sodium dodecyl sulphate; 0.2 % Coomassie Brilliant Blue G-250 dye; and 20 % glycerol) with no reducing agent. Lane 2 contains protein boiled in the presence of dye and SDS loading buffer containing 200 mM of the reducing agent 1,4-dithio-D-threitol (DTT). Lane 1 contains a dimeric disulphide bonded species. In the presence of DTT, the disulphide bonds are reduced, yielding a monomeric species.

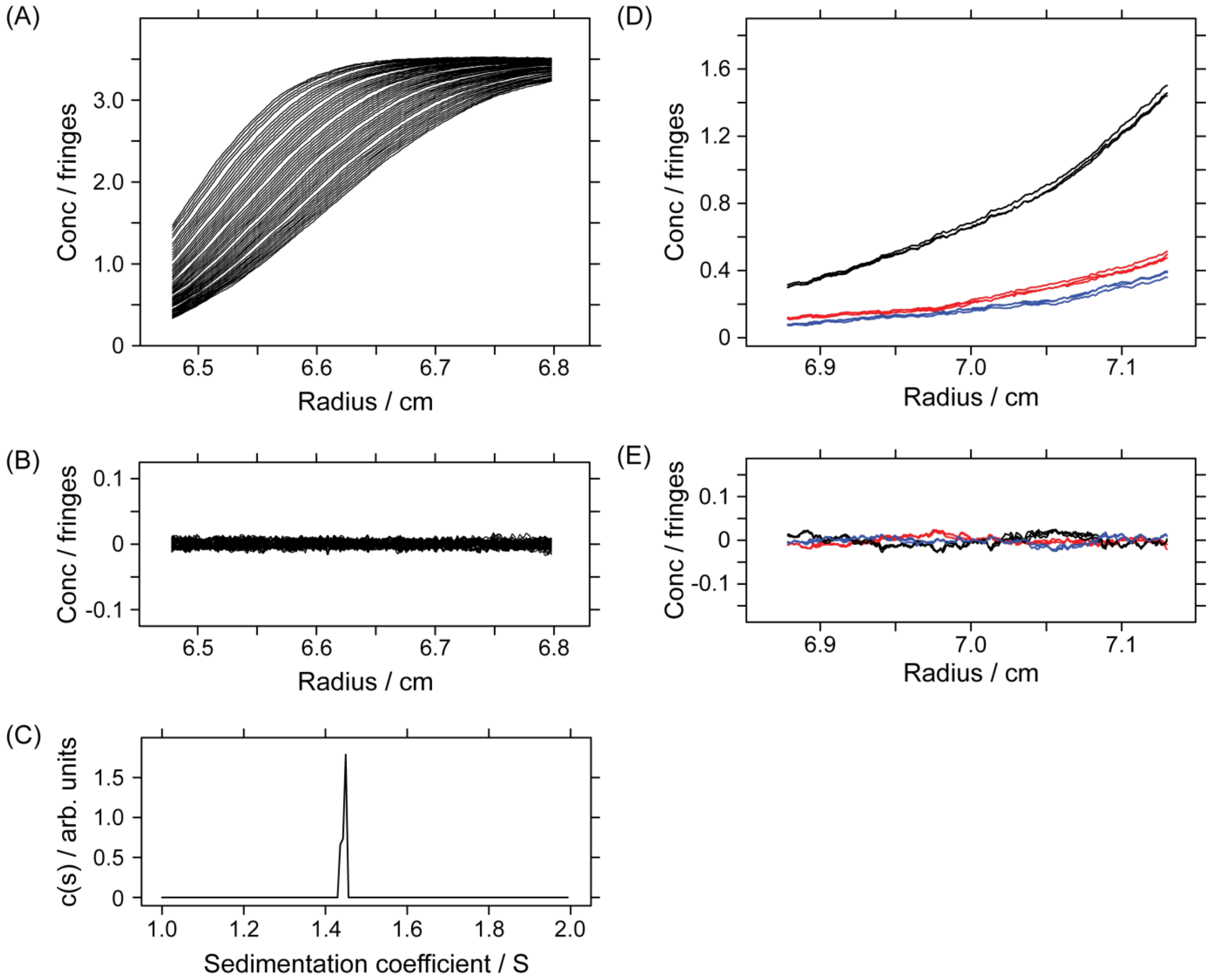

Figure S3: Analytical ultracentrifugation results for pXqua QUA1-C59S. Parts (A), (B) and (C) show typical results from a sedimentation velocity experiment at a protein concentration of 2 mg mL^{-1}: (A) concentration profiles; (B) residuals plots; and (C) a plot indicating the distribution of sedimentation coefficients necessary to fit the data; these experiments demonstrate that QUA1-C59S is present in solution as a single species. Parts (D) and (E) show typical results from a sedimentation equilibrium experiment at 25,000 rpm: (D) concentration profiles for sample concentrations of 0.5 mg mL^{-1} (blue), 1.0 mg mL^{-1} (red) and 2.0 mg mL^{-1} (black), and (E) a residuals plot; a global single species fit yielded a mean molecular weight of 12.2 ± 0.3 kDa.

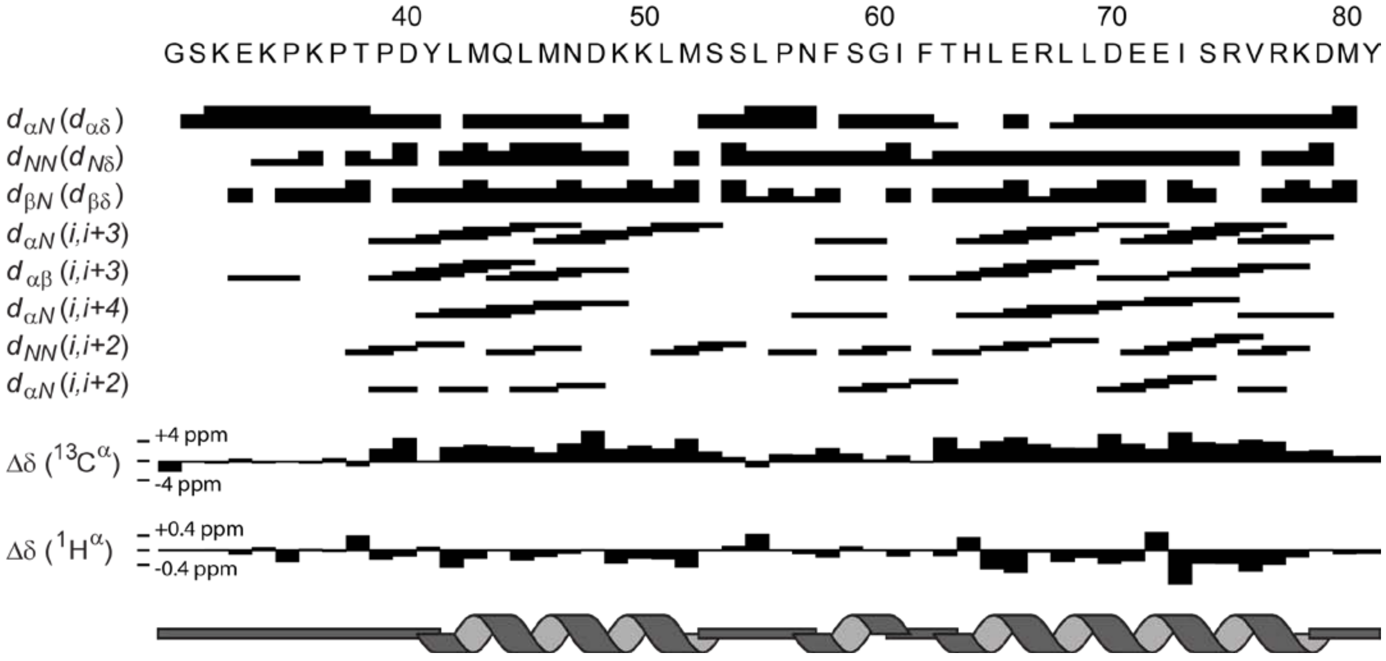

Figure S4: Summary of NMR data used to define elements of secondary structure in pXqua QUA1-C59S. The amino acid sequence is indicated, along with sequential and medium range NOE connections, secondary chemical shift values for $^{1}H^{\alpha}$ and $^{13}C^{\alpha}$ nuclei, and a schematic showing the boundaries of α-helices detected in the final solution structure.

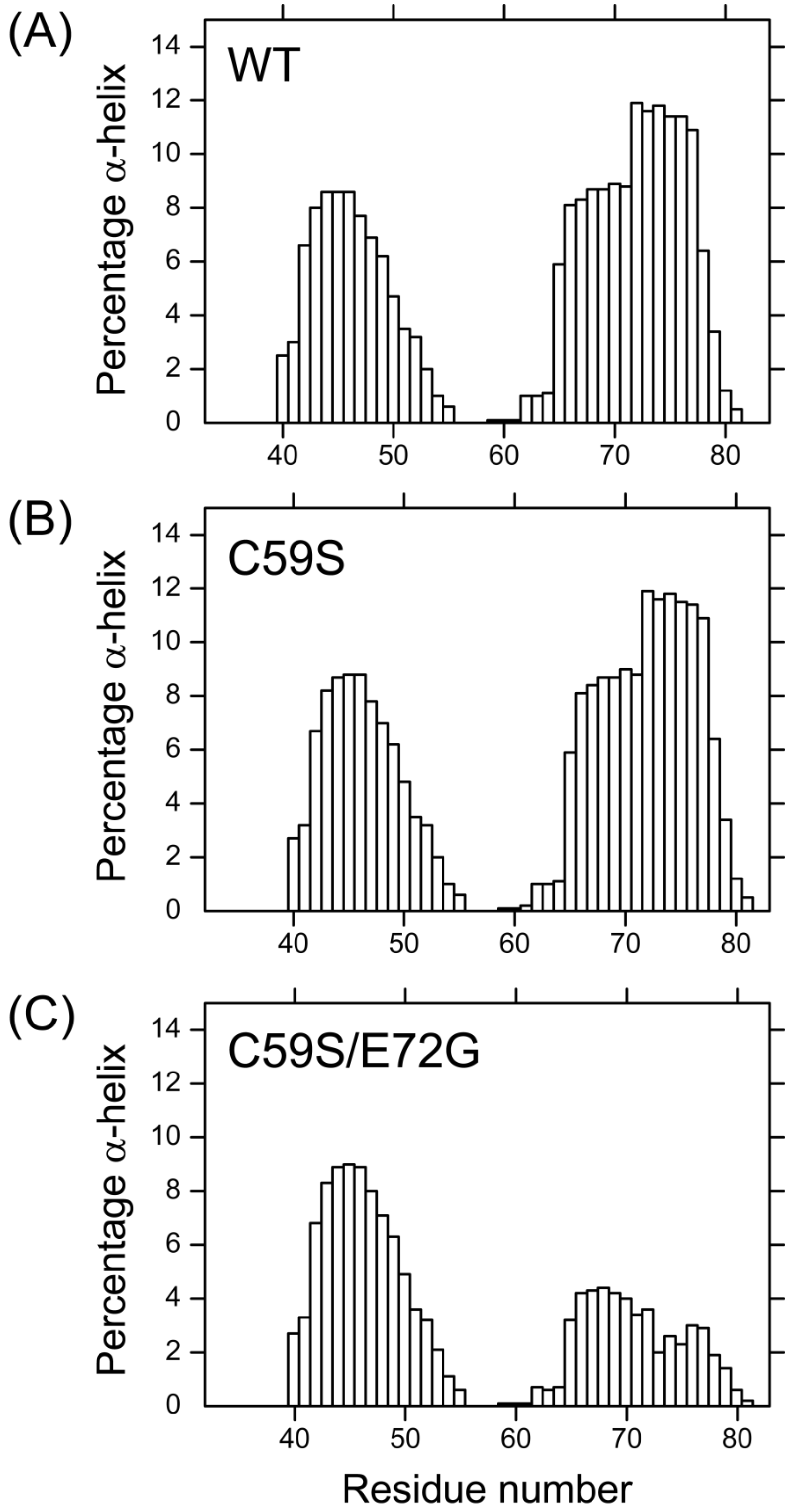

Figure S5: Per residue predictions of α-helical content made using the AGADIR server [Lacroix et al., 1998] for: (A) the wild type pXqua QUA1 sequence; (B) QUA1-C59S; (C) QUA1-C59S/E72G.

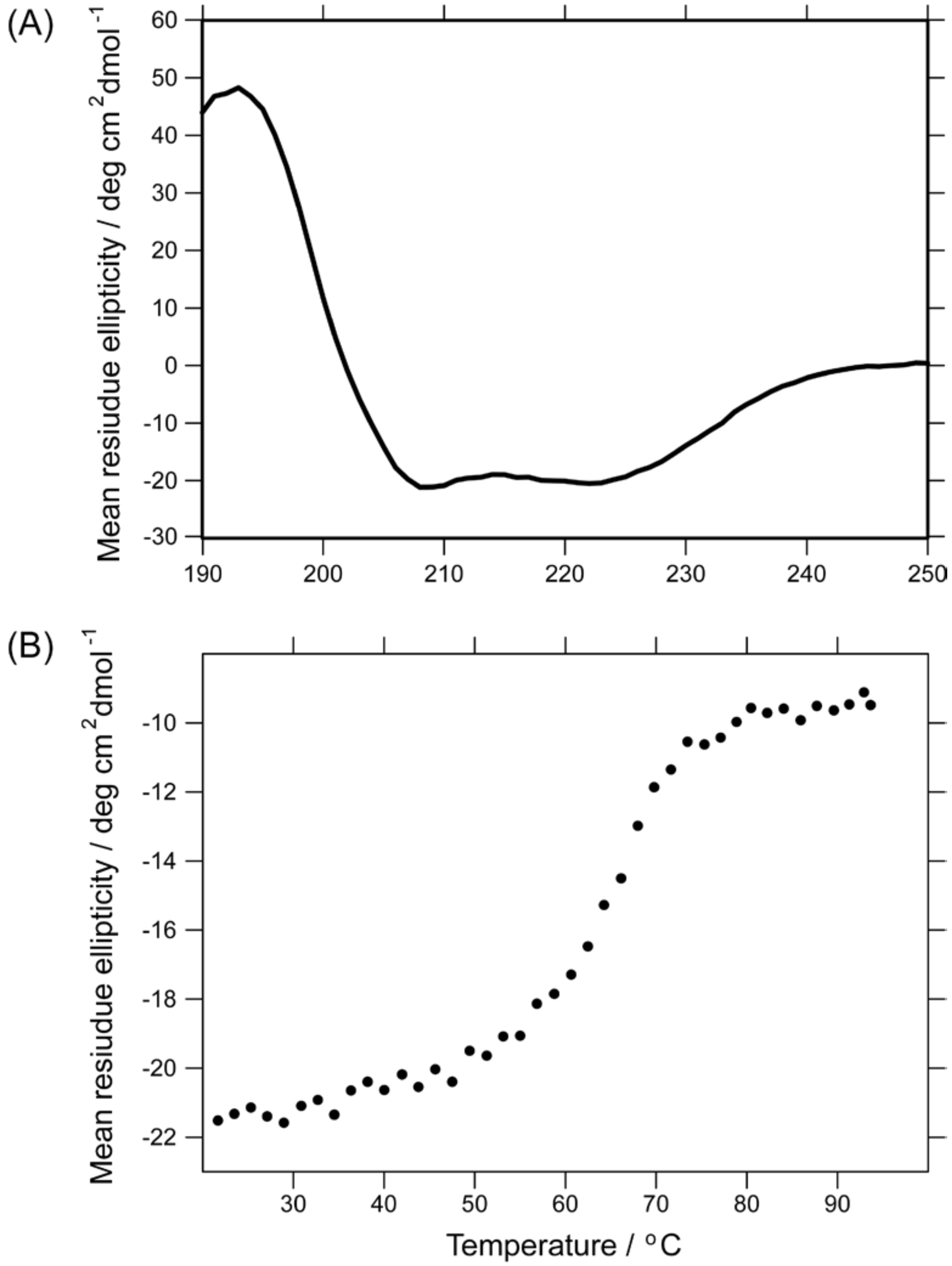

Figure S6: CD studies of the secondary structure content and thermal stability of pXqua QUA1-C59S, showing: (A) far ultraviolet CD spectrum of a 0.1 mg mL^{-1} protein sample; and (B) thermal unfolding monitored by following the CD signal at 209 nm. Using the spectrum shown in (A), the DichroWEB server [Whitmore et al, 2004] reported an α-helical content of 60 %. Fitting of the thermal denaturation profile yielded a melting temperature T_m of 65 °C.

(3) Supplementary references

Cavanagh, J., Fairbrother, W.J., Palmer, A.G., Skelton, N.J. (2006) *Protein NMR Spectroscopy: Principles and Practice.* Wiley, 2nd edn.

Kay, L.E., Torchia, D.A., and Bax, A. (1989) Backbone dynamics of proteins as studied by ^{15}N inverse detected heteronuclear NMR spectroscopy: application to staphylococcal nuclease. *Biochemistry*, **28**, 8972–8979.

Lacroix. E., Viguera, A.R., Serrano, L. (1998) Elucidating the folding problem of α-helices: local motifs, long-range electrostatics, ionic strength dependence and prediction of NMR parameters. *J. Mol. Biol.*, **284**, 173-191.

Lebowitz, J., Lewis, M.S. & Schuck, P. (2002) Modern analytical ultracentrifugation in protein science: a tutorial review. *Protein Sci.*, **11**, 2067-79.

Schuck, P. (2003) On the analysis of protein self-association by sedimentation velocity analytical ultracentrifugation. *Anal. Biochem.*, **320**, 104-124.

Vranken, W.F., Boucher, W., Stevens, T.J., Fogh, R.H., Pajon, A., Llinas, M., Ulrich, E.L., Markley, J.L., Ionides, J., and Laue, E.D. (2005) The CCPN data model for NMR spectroscopy: development of a software pipeline. *Proteins*, **59**, 687–696.

Whitmore L. & Wallace B.A. (2004). DichroWEB, an online server for protein secondary structure analyses from circular dichroism spectroscopic data. *Nuc Acid Res.*, **32**, W668-W673.
